# Dynamic contrast-enhanced MRI-based radiomics model of intra-tumoral kinetic heterogeneity for predicting breast cancer molecular subtypes

**DOI:** 10.3389/fmolb.2025.1635296

**Published:** 2025-07-18

**Authors:** Yue Cheng, Ran Ren, Yu Xu, Shaofeng Duan, Jilei Zhang, Zhongyuan Bao

**Affiliations:** ^1^ Department of Radiology, Wuxi No. 2 People’s Hospital, Jiangnan University Medical Center, Wuxi, China; ^2^ Department of Radiology, Wuxi Branch of Zhongda Hospital Southeast University, Wuxi, China; ^3^ GE Healthcare, Precision Health Institution, Shanghai, China; ^4^ Bayer Healthcare, Shanghai, China; ^5^ Department of Neurosurgery, Wuxi Institute of Neurosurgery, Wuxi, China

**Keywords:** breast cancer, subregions, kinetic heterogeneity, radiomics, dynamic contrast-enhanced magnetic resonance imaging

## Abstract

**Objectives:**

This study aims to segment intra-tumoral subregions of breast cancer based on kinetic heterogeneity using dynamic contrast-enhanced magnetic resonance imaging (DCE-MRI). It also aims to construct a radiomics model of the whole tumor and washout region to predict molecular subtypes and human epidermal growth factor receptor 2 (HER2) status.

**Methods:**

A total of 124 patients with biopsy-confirmed breast cancer were randomly divided into training and test sets in a 7:3 ratio. Quantitative analysis of breast cancer kinetic heterogeneity parameters based on DCE-MRI data was performed, dividing tumors into three subregions (Persistent, Washout, and Plateau) according to the type of voxel-level contrast enhancement. Radiomics features of the washout region and the whole tumor were extracted from the first phase of DCE-MRI enhancement. The area under the receiver operating characteristic curve (AUC) and decision curve analysis (DCA) were used to evaluate the performance of the model.

**Results:**

The radiomics model using tumor subregion (washout region) features related to kinetic heterogeneity showed the best performance for differentiating between patients with Luminal, HER2, and HER2 status, with AUC values in the train set of 0.924, 0.876, and 0.816, respectively. Exhibiting an AUC value higher than that obtained with the whole tumor and the kinetic heterogeneity parameters. DCA curves showed that the washout region model was more effective in predicting Luminal and HER2-status subtypes, compared to the whole tumor region model.

**Conclusion:**

Radiomics analysis of washout areas from high-resolution DCE-MRI breast scans has the potential to better identify molecular subtypes of breast cancer non-invasively.

## Highlights

• The tumor is divided into three subregions: persistent, washout, and plateau.
•The radiomics features of washout regions can predict molecular subtypes.
•Predicting breast cancer subtypes is more effective from the washout region.

## Introduction

Breast cancer exhibits molecular heterogeneity (Barzaman et al., 2020; Yeo and Guan, 2017; Liu et al., 2022), encompassing distinct subtypes such as Luminal A, Luminal B, HER2-enriched, and Basal-like. Each molecular subtype manifests unique pathological characteristics, dictating tailored treatment strategies and prognoses (Cheang et al., 2009; Prat et al., 2015; McCart Reed et al., 2021; Parker et al., 2009; Bitencourt et al., 2020). Luminal-type breast cancer demonstrates sensitivity to endocrine therapy but resistance to chemotherapy. HER2-positive breast cancer exhibits high invasiveness and recurrence rates, yet displays a favorable response to chemotherapy. Basal-like breast cancer presents the highest propensity for recurrence and metastasis, resulting in a poorer prognosis. Hence, accurate prediction of the molecular subtypes and HER2 status of breast cancer holds paramount importance.

Radiomics refers to the high-throughput extraction of quantitative imaging features that can reveal disease characteristics invisible to human visual assessment. By converting medical images into mineable data using advanced computational analysis, radiomics allows detection of subtle patterns that may correlate with underlying pathophysiology (Gillies et al., 2016). DCE-MRI serves as a non-invasive imaging technique that offers clear delineation of the intricate shape of breast tumors, unveils dynamic enhancements within the tumor, and elucidates the characteristics of tumor angiogenesis and heterogeneity (Yu et al., 2020). Numerous studies have demonstrated the diagnostic efficacy of the radiomics features within whole tumors derived from T1-weighted imaging (T1WI), T2-weighted imaging (T2WI), diffusion-weighted imaging (DWI), apparent diffusion coefficient (ADC) maps, and DCE-MRI or their quantitative images in predicting molecular types (Xie et al., 2019; Leithner et al., 2020; Agner et al., 2014; Chaudhury et al., 2015; Mazurowski et al., 2014). The interior of breast cancer exhibits heterogeneity and often showcases a combination of distinct kinetic dynamics that reflect the diverse biological behaviors of tumors. However, previous studies often overlook the intra-tumoral heterogeneity, leading to an inaccurate reflection of the true degree of heterogeneity within the tumor and potential deviations in patient follow-up evaluation.

Computer-aided diagnosis (CAD) is an automated software designed to analyze DCE-MRI images obtained by high spatial resolution scans, thereby reducing interpretation time (Lehman et al., 2006; Williams et al., 2007; Meeuwis et al., 2010). CAD automatically segments the tumor into three distinct color-coded subregions, corresponding to three kinetic modes: Persistent, Washout, and Plateau, while concurrently generating quantitative parameters of kinetic characteristics. Among these, the washout region is thought to primarily represent angiogenesis and contrast agent kinetic destruction in the tumor. Jin You Kim et al. observed a correlation between kinetic heterogeneity determined by CAD and disease-free survival as well as distant metastasis in breast cancer patients (Kim et al., 2020; Kim et al., 2017). However, it remains unclear whether intra-tumoral kinetic heterogeneity derived from CAD can be utilized for molecular subtype classification and whether it offers superior effectiveness compared to whole-tumor region analysis.

Therefore, this study aims to utilize the CAD method and extract radiomics features of the kinetic heterogeneity regions of breast cancer. These features will be employed to construct a radiomics model to explore its performance in predicting breast cancer molecular subtypes and HER2 status. Furthermore, these models will be compared with those constructed using features from the entire tumor.

## Materials and methods

### Patients

The study received approval from the institutional review committee. We retrospectively collected data from 269 patients who had undergone DCE-MRI scans between January 2019 and December 2021 at two tertiary medical centers (Jiangnan University Medical Center and Xishan People’s Hospital of Wuxi). The inclusion and exclusion criteria were as follows:

Inclusion criteria: (1) Preoperative breast DCE-MRI scan; (2) Pathologically proved to be primary invasive breast cancer; (3) Clinical and postoperative pathological data are complete; (4) Image quality meets the diagnostic requirements.

Exclusion criteria: (1) Surgery, radiotherapy, chemotherapy, or endocrine therapy before MRI examination (N = 65); (2) Difficulty in determining the outlined area of the lesion (N = 42); (3) Images generated by CAD are not sufficient for analysis (N = 16); (4) Patients with bilateral breast cancer (N = 22). Consequently, a total of 124 females were included (mean age 54.97 ± 12.39 years). Based on a 7:3 ratio, the patients were randomly divided into two groups: a training set (N = 87) and a test set (N = 37) for the construction of the radiomics model. Table 1 contains detailed clinical information.

**TABLE 1:** Magnetic resonance equipment and DCE scanning parameters.

Center	MR equipment	TR (ms)	TE (ms)	Reverse angle	Slice thickness (mm)	Matrix	FOV (mm)
CenterA	Siemens Magnetom Skyra 3.0T	4.5	1.7	10°	1.6	448 × 314	340 × 340
CenterB	GE SIGNA Architect 3.0T	5.2	2.0	15°	1.2	320 × 320	360 × 360

### MRI scan parameters

The breast MRI examination was performed using Siemens Magnetom Skyra 3.0T and GE SIGNA Architect 3.0T scanners. During the examination, patients were positioned prone on a dedicated breast array coil with the breasts sagging naturally. The field of view included all breast tissues, bilateral armpits, and the anterior chest wall. All breast MRI protocols included a localizing sequence followed by axial T1WI, fat-suppressed T2WI, DWI, and DCE-MRI. The DCE-MRI based on axial fat-suppressed T1-weighted three-dimensional fast low-angle shot sequence was performed before and 8 phases after gadolinium-based contrast agent (Gd-DTPA, Germany, Bayer Healthcare) administration. The contrast agent was administered at a dose of 0.1 mmol/kg (0.2 mL/kg), followed by the use of at least 15 mL of saline solution to flush the tubing. The enhanced phases lasted approximately 6–8 min, during which the patient was required to remain stationary. Table 1 shows the DCE-MRI parameters.

### Histological evaluation

Tissue samples obtained post-breast biopsy or surgery underwent collection, followed by immunohistochemical analysis using streptavidin peroxidase to determine the expression status of estrogen receptor (ER), progesterone receptor (PR), HER2, and Ki-67. Based on the expression statuses of ER, PR, Ki-67, and HER2, breast cancer was classified into four molecular sub-types: Luminal A, ER/PR positive with high PR expression (≥20%); HER2 negative, Ki-67 was low expression (<14%); Luminal B, ER and/or PR positive, HER2 negative, Ki-67 high expression (≥14%); HER2-enriched, ER and PR negative and HER-2 positive; Basal-like, ER/PR negative and HER-2 negative.

### Image processing and tumor segmentation

All images undergo preprocessing, including resampling, denoising, and enhancement, before being imported into ITK-SNAP 3.8.0 (www.itksnap.org). Two experienced radiologists (R.R. and Y.X.) performed 3D semi-automatic segmentation of the tumor on the enhanced first-phase image. To ensure segmentation accuracy, the identified tumor area undergoes further examination and correction by the senior radiologist (Y.C.). In cases of multiple lesions, only the largest cluster is included for kinetic heterogeneity analysis.

All images underwent standardized preprocessing, including resampling to isotropic 1 mm^3^ voxels using B-spline interpolation. Intensity normalization using z-score transformation relative to pectoralis muscle signal intensity. Gray-level discretization with a fixed bin width of 25 HU. Spatial normalization using affine registration to a breast MRI template. These steps ensured consistency in radiomic feature extraction across patients and scanners.

### Kinetic heterogeneity analysis

**FIGURE 1:**
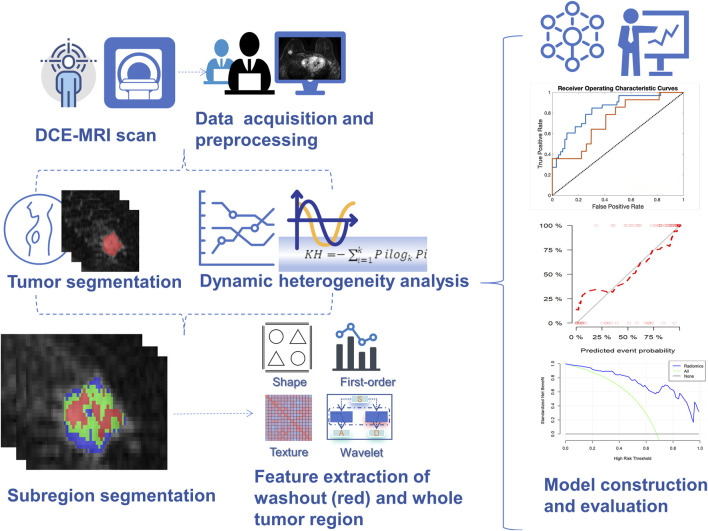
The machine learning flowchart. Firstly, high spatial resolution DCE images were acquired, and the tumor edges were manually delineated in the first phase of enhanced images. Subsequently, the tumor was classified into three sub-regions based on the kinetic heterogeneity of the tumor: persistent, washout, and plateau, which were represented in blue, red, and yellow, respectively. Finally, the radiomics features of the tumor and the red region in the tumor were extracted, and the features were downscaled to construct the diagnostic model. Diagnostic performance was calculated by the area under the receiver operating characteristic curve and decision curve analysis. *DCE,* Dynamic contrast-enhanced; *HER2,* Human epidermal growth factor receptor 2.

The process of kinetic heterogeneity analysis and radiomics is depicted in Figure 1.

The kinetic heterogeneity analysis based on breast DCE MRI utilizes an in-house CAD program written in MATLAB 8.2.0. DCE images of phase 1 + 8 and tumor segmentation masks were imported and conducted for analysis. Initially, the enhancement areas within the tumor were identified by comparing the signal intensity changes at the voxel level between the first phase of enhancement and pre-enhancement images with areas exhibiting an increase of over 50%. Subsequently, within the enhancement area, the signal intensity changes between the last phase of enhancement and the first phase of enhancement were compared. Voxel enhancement types were categorized as follows: Persistent, the signal intensity increased more than 10% from the first contrast-enhanced series (visualized in blue); Washout, the signal intensity at the last contrast-enhanced series of more than10% decreased from the first contrast-enhanced series (visualized in red); Plateau, the signal intensity change in either direction within a 10% range (visualized in yellow).

Based on the previous segmentation, peak enhancement (the highest signal intensity in the first contrast-enhanced series) and enhanced volume (the volume of lesions where the pixel value increased above the 50% threshold) are calculated. Proportions of delayed enhancement profiles were extracted. For each breast cancer case, the predominant curve type (single maximum proportion of washout, plateau, or persistent enhancement, represented by 123, respectively) and worst curve type (single most suspicious type: washout was most suspicious, followed by plateau and persistent enhancement) were determined. To quantify the degree of heterogeneity within the tumor, we used the following equation to calculate kinetic heterogeneity (a measure of heterogeneity in the proportion of tumor pixels with washout, plateau, and persistence components).

$$KH=-\sum_{i=1}^{k}Pi\log_{k}Pi$$

Pi refers to the proportion of various voxel types, and k is the number of categorical variables. The KH ranges from 0 to 1, where higher values signify greater degrees of heterogeneity. A value of 0 indicates homogeneity in the composition of the delayed enhancement area, meaning the tumor includes only one component.

The analytical method and kinetic heterogeneity parameters were similar to those used by Meeuwis et al. (2010), Kim et al. (2020).

### Radiomics analysis

#### Extraction and selection of radiomics features

Feature extraction was carried out separately for tumor regions and washout regions using Pyradiomics. Radiomics features of the tumor area (N = 1132), including shape (N = 14), first-order features (N = 234), and higher-order features (N = 884). Higher-order features include gray level co-occurrence matrix (GLCM) (N = 286), gray level dependence matrix (GLDM) (N = 182), gray level run length matrix (GLRLM) (N = 208), and gray level size zone matrix (GLSZM) (N = 208).

Radiomics features (N = 1158) of the washout region, including shape (N = 14), first-order features (N = 234), and higher-order features (N = 910). Higher-order features include gray level co-occurrence matrix (GLCM) (N = 312), gray level dependence matrix (GLDM) (N = 182), gray level run length matrix (GLRLM) (N = 208), and gray level size zone matrix (GLSZM) (N = 208).

For LASSO regularization, the optimal λ value was determined through 10-fold cross-validation in the training set, with the λ corresponding to the minimum binomial deviance selected to balance model complexity and performance. To mitigate overfitting given the high feature-to-sample ratio, we employed strict separation of training and test sets before any feature selection, and all model development was conducted exclusively on the training data (Vallieres et al., 2017). We report both training and test set performance to demonstrate generalizability, with test set performance serving as our primary outcome to avoid overfitting concerns.

#### Model construction

After feature screening was completed, we used the LASSO regression classifier to construct a prediction model for determining the validity of these selected radiomics features in predicting molecular subtypes. Firstly, the Radscore was computed by summing the selected features weighted by their coefficients. Subsequently, the Radscore from classes 0 and 1 was compared separately within the training and test groups. The performance of the model was evaluated using Receiver Operating Characteristic (ROC) analysis. Based on the Youden Index, the parameters of sensitivity, specificity, positive predictive value (PPV), negative predictive value (NPV), and accuracy were calculated. Finally, the clinical performance of the model was assessed using decision curve analysis.

### Statistical analysis

The data were randomly divided into a training set (n = 87) and a test set (n = 37). Normally distributed data are presented as mean ± standard deviation, and comparisons between the two groups were conducted using the t-test. Non-normally distributed data are presented as median (25th-75th percentile) and compared using the Mann-Whitney U test. The Kruskal-Wallis test was utilized to evaluate differences between molecular subtypes and HER2 statuses. Due to data imbalance, only Luminal, HER2-enriched, and HER2-status groups were analyzed. The diagnostic model was established using optimal features, and the performance of the test set was evaluated using the AUC. The cutoff value to maximize the Youden index was determined, and its accuracy, sensitivity, specificity, PPV, and NPV were calculated. The clinical efficacy of the model was evaluated using the DCA curve. R (version 3.6.1) was used for the analysis of variance, chi-squared test, and Kruskal-Wallis test. P < 0.05 was considered statistically significant.

## Results

### Patient characteristics

**FIGURE 2:**
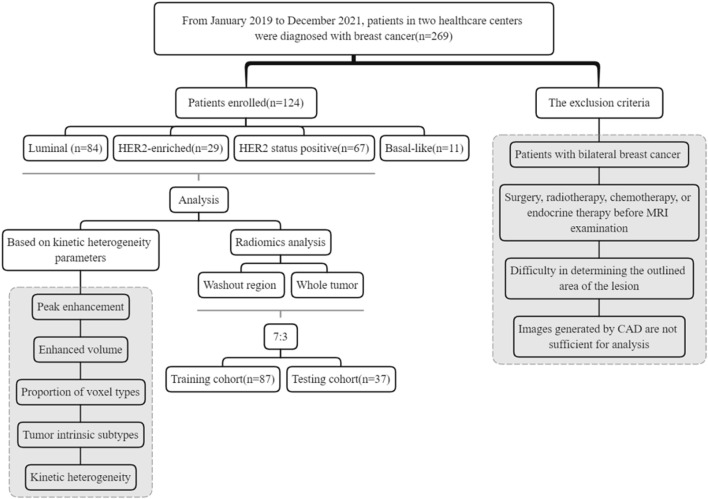
Selection flow chart of the study sample.

A total of 269 patients (mean age 54.57 ± 12.39 years) were collected from two tertiary care centers, with 124 patients ultimately included in the study (Figure 2). Among these, 86 (69.4%) cases were classified as Luminal, 29 (23.4%) cases as HER2-enriched and 11 (8.9%) cases as Basal-like subtype. HER2 status was positive in 67 patients and negative in 57 patients. The mean maximum tumor diameter was 30.27 ± 23.40 mm with a range from 5 to 79 mm. Premenopausal patients accounted for 44.35% of the total dataset. Table 2 presents the baseline characteristics of the patients.

**TABLE 2:** Demographic characteristics and dynamic heterogeneity of breast cancer subtypes.

Variable	All patients	Luminal	HER2-enriched	Basal-like	HER2 status positive
Number^a^	124	84 (67.74)	29 (23.39)	11 (8.87)	67 (54.03)
Age (y) mean ± SD	54.57 (12.39)	54.43 (13.21)	55.72 (10.32)	52.56 (10.62)	54.64 (12.27)
Maximum diameter (mm)	30.27 (23.40)	30.90 (18.30)	32.50 (38.90)	22.50 (9.47)	31.76 (29.53)
Menopausal status					
Premenopausal^a^	55 (44.35)	39 (70.91)	11 (20.00)	5 (9.09)	32 (58.18)
Postmenopausal^a^	69 (55.65)	45 (65.22)	18 (26.09)	6 (8.70)	35 (50.72)
kinetic heterogeneity parameters					
Peak enhancement	21.02 (25.00)	20.45 (24.51)	27.25 (29.33)	9.01 (4.43)	26.44 (28.14)
Enhanced volume	27,785.60 (70,002)	17,741.87 (30,097.17)	60,047.79 (130,892.6)	19,428.36 (28,987.16)	30,945.10 (83,868.02)
Persistent component (%)	41.30 (22.50)	40.90 (22.40)	41.50 (24.50)	43.30 (20.00)	36.40 (22.30)
Plateau component (%)	33.80 (19.80)	33.80 (19.00)	35.90 (24.50)	28.10 (9.00)	37.40 (23.40)
Washout component (%)	24.90 (17.60)	25.30 (17.60)	22.50 (17.50)	28.60 (18.60)	26.20 (18.60)
Kinetic heterogeneity	0.90 (0.24)	0.91 (0.24)	0.85 (0.24)	0.97 (0.16)	0.88 (0.24)
Predominant type 1^a^	68 (54.84)	43 (51.19)	18 (62.07)	7 (63.64)	32 (47.76)
Predominant type 2^a^	25 (20.16)	18 (21.43)	6 (20.69)	1 (9.09)	17 (25.37)
Predominant type 3^a^	31 (25.00)	23 (27.38)	5 (17.24)	3 (27.27)	18 (26.87)
Worst type^a^	124 (100.000)	84 (100.000)	29 (100.000)	11 (100.000)	67 (100.000)

^a^ Data are percentages mean. Unless otherwise noted, numbers in parentheses are values ± standard deviations.

*HER2*, Human epidermal growth factor receptor 2.

### Analysis of kinetic heterogeneity parameters

Table 2 lists the heterogeneity values of breast cancer subtypes. The average kinetic heterogeneity of all breast cancers was 0.898 ± 0.235. The ROC analysis results based on the kinetic parameters are presented in Table 3. In differentiating between Luminal and HER2-enriched subtypes, the highest AUCs were achieved by enhanced volume, which were 0.582 and 0.612, respectively. For identifying HER2 status, peak enhancement yielded the highest AUC, which was 0.633.

**TABLE 3:** ROC curve of dynamic heterogeneity parameters.

Variable	Sensitivity	Specificity	PPV	NPV	AUC
Luminal					
Peak enhancement	0.571	0.55	0.727	0.379	0.497
Enhanced volume	0.821	0.4	0.742	0.516	0.582
Persistent component	0.524	0.6	0.733	0.375	0.513
Plateau component	0.845	0.3	0.717	0.480	0.543
Washout component	0.738	0.4	0.721	0.421	0.522
Kinetic heterogeneity	0.357	0.775	0.769	0.365	0.546
Predominant	0.488	0.625	0.732	0.368	0.559
HER2-enriched					
Peak enhancement	0.586	0.695	0.370	0.846	0.546
Enhanced volume	0.482	0.821	0.452	0.839	0.612
Persistent component	0.172	0.926	0.417	0.786	0.505
Plateau component	0.345	0.842	0.400	0.808	0.539
Washout component	0.483	0.758	0.378	0.828	0.556
Kinetic heterogeneity	0.448	0.779	0.382	0.822	0.575
Predominant	1	0	0.234	-	0.442
HER2-Status positive					
Peak enhancement	0.507	0.772	0.723	0.571	0.631
Enhanced volume	0.552	0.667	0.661	0.559	0.583
Persistent component	0.493	0.754	0.702	0.558	0.633
Plateau component	0.269	0.930	0.818	0.520	0.540
Washout component	0.597	0.544	0.606	0.534	0.538
Kinetic heterogeneity	0.373	0.772	0.658	0.512	0.557
Predominant	0.522	0.632	0.625	0.529	0.567

*HER2*, Human epidermal growth factor receptor 2.

### Performance of the prediction model

**FIGURE 3:**
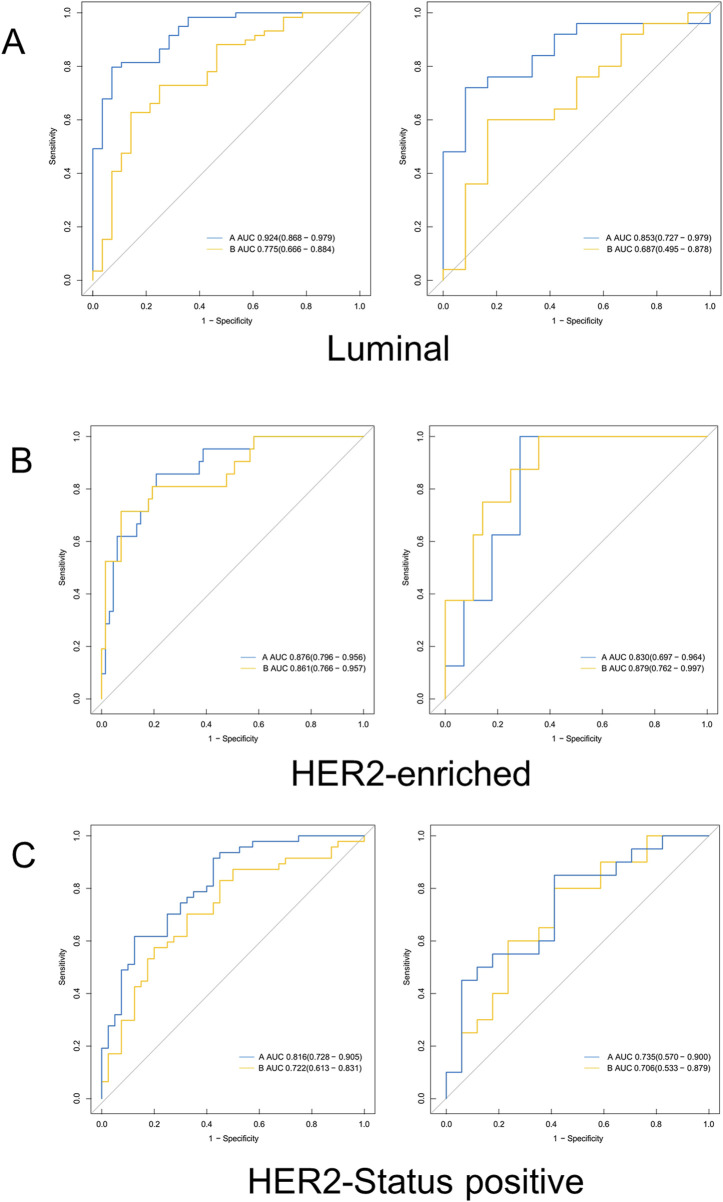
Receiver operating characteristic curves derived from the 3D washout region (blue line) related to dynamic kinetic parameters and whole tumor (yellow line) for **(A)** Luminal, **(B)** HER2-enriched, **(C)** HER2-Status positive. *HER2,* Human epidermal growth factor receptor 2; *AUC,* Area under the working characteristic curve.

Two radiomics models were constructed based on features from the whole-tumor region and the washout region, respectively. Table 4 and Figure 3 show the AUC values of the machine learning models for predicting molecular subtypes based on features extracted from the whole tumor area and the washout region in both the training set and test set.

**TABLE 4:** In the training and test set, the performance of the machine learning model of molecular subtypes is predicted based on the radiomics features of the washout region and the whole tumor region.

Variable		Sensitivity	Specificity	PPV	NPV	AUC
Whole-tumor						
Luminal AB	Train	0.729	0.750	0.86	0.568	0.775
	Test	0.720	0.500	0.75	0.462	0.687
HER2-enriched	Train	0.810	0.806	0.567	0.931	0.861
	Test	0.750	0.857	0.600	0.923	0.879
HER2-status positive	Train	0.702	0.750	0.767	0.682	0.722
	Test	0.550	0.765	0.733	0.591	0.706
Washout region						
Luminal AB	Train	0.814	0.893	0.941	0.694	0.924
	Test	0.760	0.833	0.905	0.625	0.853
HER2-enriched	Train	0.857	0.791	0.563	0.946	0.876
	Test	0.625	0.786	0.455	0.880	0.803
HER2-status positive	Train	0.702	0.675	0.717	0.659	0.816
	Test	0.700	0.588	0.667	0.625	0.735

*HER2,* Human epidermal growth factor receptor 2; *PPV,* positive predictive value; *NPV,* negative predictive value; *AUC,* area under the working characteristic curve.

For Luminal type prediction, the model performance based on the washout region (AUC = 0.924, 95% CI: 0.876–0.972) was much higher than that based on the whole tumor (AUC = 0.775) in the training set. Similarly, the model performance based on the washout region (AUC = 0.853, 95% CI: 0.742–0.964) was much higher than that based on the whole tumor (AUC = 0.687) in the test set.

In terms of HER2-enriched type prediction, the model performance based on the washout region (AUC = 0.876) was slightly higher than that based on the entire tumor (AUC = 0.861) in the training set. However, in the test set, the performance based on the washout area (AUC = 0.830) was slightly lower than the performance based on the whole tumor (AUC = 0.879).

For HER2-status positive type prediction, the model performance (AUC = 0.816) based on the washout region is higher than that based on the whole tumor (AUC = 0.722) in the training set, and the model performance based on the washout region (AUC = 0.735) is slightly higher than that based on the whole tumor (AUC = 0.706) in the test set.

**FIGURE 4:**
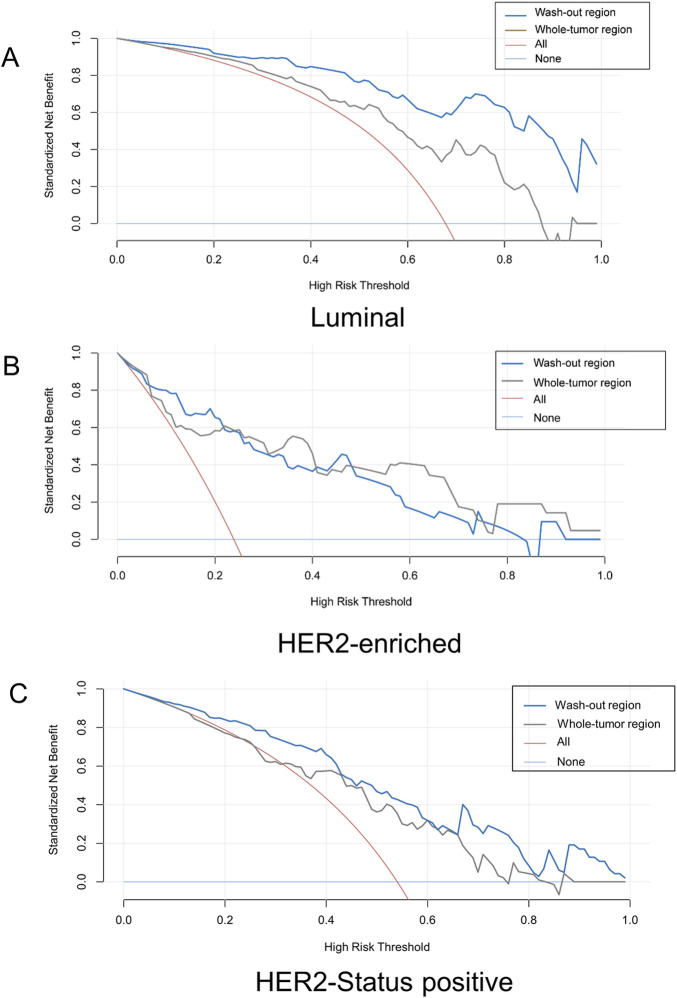
Decision curve analysis curves derived from the 3D washout region (blue line) related to dynamic kinetic parameters and whole tumor (gray line) for **(A)** Luminal, **(B)** HER2-enriched, **(C)** HER2-Status positive. *HER2,* Human epidermal growth factor receptor 2.

DCA for Luminal, HER2-enriched subtypes, and HER2-status positive is presented in Figure 4. The DCA curve showed that the clinical efficacy of the model based on the washout region was higher than that of the whole-tumor area in predicting Luminal and HER2-status positive statuses. For predicting HER2-enriched, the clinical efficacy of the model based on the washout region was higher than that of the whole-tumor region when the high-risk threshold fell within a certain range.

## Discussion

This study introduces a novel approach utilizing kinetic heterogeneity analysis based on DCE-MRI to segment intra-tumoral subregions into three distinct categories: persistent enhancement, washout, and plateau. Subsequently, radiomics models were developed leveraging features extracted from both the washout region and the whole tumor region. These models aim to predict molecular subtypes and HER2 status in breast cancer and assess their clinical efficacy. Our findings indicate that the diagnostic efficacy of the radiomics model based on the washout region within the tumor surpasses that of the whole-tumor model, particularly in predicting Luminal subtypes. Notably, the diagnostic performance is highest with an AUC of 0.924 for the training set and 0.853 for the test set.

Previous studies have demonstrated the clinical significance of radiomics analysis in distinguishing benign and malignant breast lesions, as well as in differentiating various histopathological types, grades, and stages of breast tumors (Braman et al., 2017; Li et al., 2016; Zhou J. et al., 2020; Mao et al., 2020; Ma et al., 2018; Liu et al., 2019). However, most of these studies focused on constructing radiomics models using the entire tumor area. In contrast, our approach involves analyzing the internal tumor heterogeneity and segmenting tumors based on kinetic heterogeneity. Segmenting and modeling tumors into sub-regions is crucial for accurately capturing internal heterogeneity. Breast cancer masses exhibit high heterogeneity, with various features mixed within the tumor. Focusing on the entire tumor region dilutes this heterogeneity and may weaken model performance. In contrast, the washout region, which best reflects tumor malignancy, allows for a more precise analysis of tumor heterogeneity.

The washout pattern is strongly associated with tumor angiogenesis and vascular permeability (Kuhl et al., 1999). Rapid contrast agent washout reflects immature, leaky tumor vasculature with high endothelial proliferation, characteristic of more aggressive tumors (Knopp et al., 1999). From a microenvironment perspective, washout regions correlate with areas of hypoxia and extracellular matrix remodeling, which promote epithelial-mesenchymal transition and metastatic potential (Li and Padhani, 2012; Pickles et al., 2005). At the molecular level, washout regions show elevated expression of vascular endothelial growth factor (VEGF) and other pro-angiogenic factors that drive the HER2 and basal-like phenotypes.

Recently, alternative data-driven methods have been employed to delineate tumor subregions, such as pharmacokinetic models and habitat analysis models (Liu et al., 2020; Kim et al., 2016; Xu et al., 2024). Studies have demonstrated that the Tofts model can assess pharmacokinetic parameters and analyze the vascular permeability of tumors (Zhou X. et al., 2020; Mouawad et al., 2020; Ioannidis et al., 2019). While pharmacokinetic models like the Tofts model provide quantitative parameters of vascular permeability, they require high temporal resolution (5–10 s) acquisitions, which are not routinely available in clinical practice (Sourbron and Buckley, 2013). Our approach using standard high spatial resolution DCE-MRI makes the technique more clinically feasible. Zhou X et al. diagnosed breast cancer and predicted molecular subtypes using pharmacokinetic dynamically-enhanced (PK-DCE) MRI in the Tofts model (Zhou X. et al., 2020). They found PK-DCE MRI to be superior in breast cancer diagnosis but less effective in predicting molecular subtypes, achieving AUCs of 0.71 ∼ 0.77 for predicting the Luminal subtype and 0.61 ∼ 0.68 for predicting the HER2-enriched subtype. In comparison, our model achieved higher AUCs of 0.924 for Luminal and 0.879 for HER2-enriched subtypes.

In recent years, with the rapid advancement of artificial intelligence, researchers have explored methods such as habitat analysis and unsupervised learning to be used in breast cancer (Sun et al., 2022; Twellmann et al., 2008; Cho et al., 2022). Jia Wu et al. performed molecular subtype prediction by clustering analysis of quantitative image features (Wu et al., 2017). Ming Fan et al. attempted tumor classification based on values of Time to Peak, Peak Enhancement Ratio, and Kinetic Pattern Clustering (Fan et al., 2018). They also applied unsupervised clustering analysis to decompose time series curves at the pixel level into specific regions for plasma input, fast-flow dynamics, and slow-flow dynamics (Fan et al., 2019). Their results consistently showed that intra-tumor radiomics analysis improves the predictive performance compared to the whole tumor approaches, with AUCs ranging from 0.74 to 0.88. Zhang et al. similarly reported significant diagnostic enhancements, particularly for Luminal subtype analysis (Zhang et al., 2022), aligning closely with our findings. These studies suggest that the model based on subregional radiomics features for predicting the Luminal subtype may serve as a more valuable imaging marker.

Although unsupervised clustering methods can identify tumor subregions, the biological interpretation of these clusters may be ambiguous (Wu et al., 2017; Leithner et al., 2018). Our method’s clear delineation into three physiologically meaningful regions (persistent, washout, plateau) provides more clinically intuitive results. The CAD software used in our study offers automated quantitative analysis with minimal user intervention, enhancing reproducibility in clinical settings compared to more complex AI methods that may require specialized expertise.

Our research has several limitations. Firstly, it is a retrospective study conducted at two local hospitals, which may introduce selection bias and geographical limitations in the study population. To enhance generalizability and interpretability before clinical application, data should be collected from a prospective multicenter standardized database. Secondly, the study did not account for the effects of the menstrual cycle and hormone levels, nor did it consider the tumor microenvironment. Thirdly, our study excluded TNBC cases due to limited sample size, which represents a significant limitation given the clinical importance of this aggressive subtype. TNBC typically demonstrates more rapid and pronounced washout kinetics than other subtypes (Youk et al., 2012), suggesting our model might perform differently for these cases. Future studies should specifically evaluate whether washout region features have particular value in characterizing TNBC, which could help address the current lack of targeted therapies for this subtype (Bianchini et al., 2016). Additionally, we aim to integrate multi-parametric and multi-modal imaging with genomics and genomics analysis to develop higher-dimensional, multi-scale, and more efficient diagnostic models.

In conclusion, this study explores a novel non-invasive tumor segmentation method using high spatial resolution DCE-MRI images. It analyzes tumor kinetic heterogeneity by segmenting the sub-tumor region into three distinct areas. Features from the washout region within the tumor were extracted to construct a radiomics model for predicting molecular subtypes. The findings indicate that radiomics features of the washout region derived from kinetic heterogeneity analysis may serve as predictive markers for molecular subtypes, particularly in predicting Luminal subtypes. Visualizing tumor heterogeneity in this way could potentially enhance treatment precision and personalization for breast cancer patients.

## Data Availability

The dataset used in this study is available through an institutional review application for academic non-commercial use only. Requests to access the datasets should be directed to Yue Cheng, cy624717562@126.com.

## Abbreviations

DCE-MRI, Dynamic contrast-enhanced magnetic resonance imaging; HER2, Human epidermal growth factor receptor 2; AUC, Area under the receiver operating characteristic curve; DCA, Decision curve analysis; T1WI, T1-weighted imaging; T2WI, T2-weighted imaging; DWI, Diffusion-weighted imaging; ADC, Apparent diffusion coefficient; CAD, Computer-aided diagnosis; ER, Estrogen receptor; PR, Progesterone receptor; GLCM, Gray level co-occurrence matrix; GLDM, Gray level dependence matrix; GLRLM, Gray level run length matrix; GLSZM, Gray level size zone matrix; mRMR, Minimum redundancy maximum relevance; LASSO, Least absolute shrinkage and selection operator; ROC, Receiver operating characteristic; PPV, Positive predictive value; NPV, Negative predictive value; PK-DCE, Pharmacokinetic dynamically-enhanced.
